# Evaluation of the SARS-CoV-2 Inactivation Efficacy Associated With Buffers From Three Kits Used on High-Throughput RNA Extraction Platforms

**DOI:** 10.3389/fcimb.2021.716436

**Published:** 2021-09-16

**Authors:** Ruth E. Thom, Lin S. Eastaugh, Lyn M. O’Brien, David O. Ulaeto, James S. Findlay, Sophie J. Smither, Amanda L. Phelps, Helen L. Stapleton, Karleigh A. Hamblin, Simon A. Weller

**Affiliations:** CBR Division, Dstl Porton Down, Salisbury, United Kingdom

**Keywords:** SARS-CoV-2, high throughput, PCR, biosafety, laboratory-acquired infection, clinical diagnosis

## Abstract

Rapid and demonstrable inactivation of SARS-CoV-2 is crucial to ensure operator safety during high-throughput testing of clinical samples. The inactivation efficacy of SARS-CoV-2 was evaluated using commercially available lysis buffers from three viral RNA extraction kits used on two high-throughput (96-well) RNA extraction platforms (Qiagen QIAcube HT and the Thermo Fisher KingFisher Flex) in combination with thermal treatment. Buffer volumes and sample ratios were chosen for their optimised suitability for RNA extraction rather than inactivation efficacy and tested against a representative sample type: SARS-CoV-2 spiked into viral transport medium (VTM). A lysis buffer mix from the MagMAX Pathogen RNA/DNA kit (Thermo Fisher), used on the KingFisher Flex, which included guanidinium isothiocyanate (GITC), a detergent, and isopropanol, demonstrated a minimum inactivation efficacy of 1 × 10^5^ tissue culture infectious dose (TCID)_50_/ml. Alternative lysis buffer mixes from the MagMAX Viral/Pathogen Nucleic Acid kit (Thermo Fisher) also used on the KingFisher Flex and from the QIAamp 96 Virus QIAcube HT Kit (Qiagen) used on the QIAcube HT (both of which contained GITC and a detergent) reduced titres by 1 × 10^4^ TCID_50_/ml but did not completely inactivate the virus. Heat treatment alone (15 min, 68°C) did not completely inactivate the virus, demonstrating a reduction of 1 × 10^3^ TCID_50_/ml. When inactivation methods included both heat treatment and addition of lysis buffer, all methods were shown to completely inactivate SARS-CoV-2 inactivation against the viral titres tested. Results are discussed in the context of the operation of a high-throughput diagnostic laboratory.

## Highlights

- To date, there have been few publications on the inactivation of severe acute respiratory syndrome coronavirus-2 (SARS-CoV-2) in the diagnostic context.
- This publication adds to the published knowledge and helps laboratories that do not have microbiological containment facilities (biosafety level (BSL) 3 or above) and therefore are not able to perform detailed experimental research in this area and assess the safety of their SARS-CoV-2 diagnostic processes.
- The findings of the paper show that a combination of chemical treatments and/or physical methods such as the application of heat are required to inactivate SARS-CoV-2 in nasal swab samples and are in concordance with a similar paper from this group on the inactivation of Ebola virus in diagnostic samples.
- This will support laboratories and reduce the likelihood of laboratory-acquired infections.

## Introduction

Severe acute respiratory syndrome coronavirus-2 (SARS-CoV-2) belongs to the *Coronaviridae* family and is the causative agent of the respiratory illness, coronavirus disease 2019 (COVID-19) (Gorbalenya et al., 2020). The enveloped positive-sense single-stranded RNA virus was first discovered in early 2020 after a cluster of viral pneumonia cases of unknown cause were reported in the Hubei Province of China (Wu et al., 2020). The virus is highly contagious in humans, and in March 2020, the WHO declared a global pandemic (Chen, 2020).

Diagnostic testing is critical in the fight against the COVID-19 pandemic (Patel et al., 2020), not just for patients displaying symptoms but also for asymptomatic carriers and pre-symptomatic patients (Shental et al., 2020). SARS-CoV-2 has been classified in the United Kingdom as a Hazard Group (HG) 3 pathogen by the Advisory Committee on Dangerous Pathogens (ACDP), meaning that this virus must be handled under Containment Level (CL) 3 conditions [biosafety level (BSL) 3]. However, guidance from the WHO (World Health Organization 2020) and Public Health England, United Kingdom (Public Health England, 2020), has permitted non-propagative diagnostic testing to be carried out at CL 2 with non-inactivated samples being handled within a Class 1 microbiology safety cabinet.

Real-time reverse transcriptase polymerase chain reaction (RT-PCR) is the gold standard test for the detection of SARS-CoV-2 from nasopharyngeal swab samples (Tahamtan and Ardebili, 2020). Inactivation of viral pathogens prior to PCR is typically carried out at the same time as extraction of viral nucleic acids from samples, with chemical or physical methods employed. Typically buffers provided in nucleic acid extraction kits contain chaotropic salts, solvents, and detergents to lyse the virus. Guanidinium salts, such as guanidinium isothiocyanate (GITC), are chaotropic agents found in many lysis buffers, which in some cases have been demonstrated to inactivate viral pathogens, including alphaviruses, flaviviruses, filoviruses, and a bunyavirus (Blow et al., 2004; Ngo et al., 2017). Other reports suggest that a combination of a GITC containing extraction buffer (such as Qiagen AVL) and a solvent (such as ethanol) is required for the inactivation of viruses such as Ebola virus (Smither et al., 2015) and Middle East respiratory syndrome coronavirus (MERS-CoV) (Kumar et al., 2015). Detergents such as Tween, sodium dodecyl sulphate (SDS), and Triton X-100 have also been shown to disrupt viral envelopes and reduce viral titres (Mayo and Beckwith, 2002; van Kampen et al., 2017; Patterson et al., 2020), with a combination of the GITC-based reagent (Buffer AVL) and Triton X-100 having been reported to inactivate Ebola virus (Burton et al., 2017). Physical processes such as heat can also be incorporated in the nucleic acid extraction workflow and can have an inactivation effect. Some reports suggest that the application of heat alone can inactivate SARS-CoV, MERS-CoV, and SARS-CoV-2 following a heat regimen of 65°C for at least 15 min (Darnell et al., 2004; Leclercq et al., 2014; Kim et al., 2020).

Since the pandemic was declared, United Kingdom’s Defence Science and Technology Laboratory (Dstl) and British military clinicians have set up the Defence COVID Laboratory (DCL), which has been awarded an extension to scope (under ISO17025) for the provision of a SARS-CoV-2 PCR test by the United Kingdom Accreditation Service (UKAS). The DCL analyses samples from UK military units and operates two automated high-throughput RNA extraction platforms (Qiagen QIAcube HT and the Thermo Fisher KingFisher Flex). In this study, conducted entirely under CL 3 laboratory conditions (BSL 3), we report the inactivation efficacy of SARS-CoV-2 by buffers from three commercially available kits used on these two platforms. Buffer volumes and ratios were chosen for their suitability for RNA extraction (following manufacturer’s instructions) rather than their potential inactivation efficacy; however, in doing so, we have further investigated the inactivation efficacy of combinations of GITC containing buffers, solvents, and/or detergents with and without an additional heat inactivation step. We provide evidence to support protocols for the inactivation of SARS-CoV-2 and the safe use of clinical samples in downstream RT-PCR in high-throughput diagnostic laboratories.

## Methods

### Virus Strains, Cell Culture, and Enumeration

All virus manipulations were carried out using the SARS-CoV-2 England 2 strain (GISAID reference EPI_ISL_407073), provided by Public Health England. Virus stock was propagated in Vero C1008 cell, harvested at day 3 and clarified by centrifugation at 350 × *g* for 15 min (Sigma 3-16K centrifuge). Viral stocks were concentrated by centrifugation at 11,000 × *g* for 3 h at 4°C to achieve 1 × 10^8^ tissue culture infectious dose (TCID)_50_/ml and stored at −80°C.

All cell cultures were carried out using confluent monolayers of Vero C1008 cells (European Collection of Authenticated Cell Cultures [ECACC], United Kingdom; catalogue no. 85020206) maintained in Dulbecco’s minimal essential medium (DMEM; Sigma, United Kingdom) supplemented with 10% foetal calf serum, 1% l-glutamine, and 1% penicillin–streptomycin (Sigma, United Kingdom) and incubated at 37°C in a 5% CO_2_ environment. Prior to virus being added to cell monolayers, 10% DMEM was replaced with Leibovitz’s L-15 (to buffer for the lack of CO_2_ at CL 3), supplemented as described for DMEM, with the exception of 2% foetal calf serum, and incubated at 37°C.

Viral enumeration (for determining starting concentrations and measuring reductions in concentrations post-inactivation) was carried out by an end-point TCID_50_ assay (Piercy et al., 2010). In brief, Vero C1008 cells were prepared in 96-well microtitre plates to achieve confluent monolayers on the day of assay. To all wells of column 1 of the plate, 100 µl of test sample was added. From column 1, 20 µl of sample was transferred sequentially across the plate to achieve a 10-fold serial dilution to column 9. Cells in columns 11 and 12 were left in tissue culture medium (TCM) as controls. Plates were incubated in a humidified atmosphere for 3–4 days at 37°C, after which they were scored for cytopathic effect (CPE) by microscopic observation. The TCID_50_ value was calculated by the method of Reed and Muench (1938). Mean values were calculated as the geometric mean.

### Viral Inactivation

Buffers and reagents from three different RNA extraction kits were assessed to determine inactivation of SARS-CoV-2 (**Table 1**). The composition of these initial reagents and their suitability for extraction of SARS-CoV-2 RNA from clinical samples was determined based on manufacturers’ protocols and after discussions with each manufacturer.

**Table 1:** Protocols tested for assessing inactivation using lysis buffers.

Manufacturer, RNA extraction kit, platform	Reagents (volume/sample)	Active virucidal components^*^	Reagent:sample ratio
Qiagen,	ACL buffer (190 µl)	GITC 30% to <50%	1.6:1
QIAamp 96 Virus QIAcube HT Kit			
(Cat #: 57731),	ATL buffer (100 µl)	1% to <3% SDS	
Qiagen QIAcube HT.			
*(Referred to here as Qiagen protocol)*	Proteinase K (20 µl)		
	Carrier RNA (5 µl)		
	MS2 (10 µl)		
Thermo Fisher,	Lysis binding buffer (350 µl)	GITC 55%–80% <0.001% acrylamide	3.8:1
MagMAX Pathogen RNA/DNA kit		Zwittergent	
(Cat #: 4462359),			
KingFisher Flex.			
*(Referred to here as* MagMAX *Protocol 1)*	Isopropanol (300 µl)	100% 2-propanol	
	Carrier RNA (2 µl)		
	Water (100 µl)		
	MS2 (10 µl)		
Thermo Fisher,	Lysis binding buffer (265 µl)	GITC 55%–80% <0.001% acrylamide	1.4:1
MagMAX viral/pathogen nucleic acid isolation kit		Zwittergent	
(Cat #: A48310),			
KingFisher Flex.			
*(Referred to here as* MagMAX *Protocol 2)*	Proteinase K (5 µl)		
	^†^Water (magnetic beads) (10 µl)		
	MS2 (10 µl)		

^*^As identified directly from components or manufacturer information or inferred from the associated MSDS.

^†^Water was used to replace the magnetic beads, as the washing steps described below would not remove the beads, and the beads interfered with the read-out of the TCID_50_ assay.

GITC, Guanidinium thiocyanate; SDS, Sodium dodecyl sulphate.

The inactivation efficacy of each lysis buffer was evaluated with and without the inclusion of a heat step. **Table 1** summarises the components and volumes used for each lysis buffer preparation. MS2 bacteriophage (10^6^ plaque-forming unit (PFU)/ml) was added to each lysis buffer preparation as an internal control in the DCL and was therefore included in these experiments. Test samples for each experiment were set up in triplicate, and each experiment was performed on at least three separate occasions.

Viral transport medium (VTM; EO Labs, United Kingdom) was inoculated with SARS-CoV-2 to achieve a starting concentration of 5 × 10^6^ TCID_50_/ml for all experiments. To the lysis buffer preparations, 200 µl of virus in VTM was added; the samples were briefly vortexed and incubated for 10 min at room temperature. For heat-treated samples, the tubes were incubated for 25 min in a heat block (Eppendorf ThermoMixer C heat) set at 75°C. Laboratory tests showed that this was the temperature setting required for this individual heat block to heat and maintain the samples at 68°C for 15 min. Heat steps were carried out after the addition of virus to either lysis buffer reagents or an equivalent volume of TCM, to assess the effect of viability following heat in the presence or absence of reagents. Further controls included sham-inactivated virus, where appropriate volume of TCM replaced the lysis buffer reagents and negative controls consisting of VTM only were added to lysis buffer reagents to assess the effect of the reagents on cell monolayers.

After inactivation (with or without heat treatment), all samples and controls were centrifuged at 6,000 × *g* for 5 min in a microcentrifuge (Hermle Microlitre Centrifuge Z 160 M), and the supernatant was discarded and replaced with 1 ml of TCM. This step was required to dilute the chemical components that would otherwise cause toxicity in the cell culture-based enumeration assay. Although virus pellets were not visible, this method is known to pellet virus with appropriate efficiency, as demonstrated by virus recovery in positive controls and is similar to methods used successfully in previous studies (Smither et al., 2016). In experiments (data not shown), this step was shown to be required four times for the Qiagen reagents and two times for the KingFisher reagents in order to remove all traces of the inactivation chemicals from the sample and to avoid toxicity during cell culture. After the final wash, the pellets were re-suspended in 1 ml of TCM. Controls in each experiment were washed the same number of times as required by the reagent being evaluated.

### Post-Inactivation Viral Viability Assays

To quantify and determine the viability of the virus following inactivation, the samples were enumerated by the TCID_50_ end-point dilution assay described above; and the remaining sample underwent three rounds of serial passage in tissue culture flasks for a secondary confirmation of viral inactivation. In brief, all of the remaining samples (approx. 180 µl) were added to confluent monolayer of Vero C1008 cells in a 12.5-cm^2^ tissue culture flask. Flasks were incubated in a humidified atmosphere for 3–4 days after which presence or absence of cytopathic effect was recorded. A total of three passages were performed, and CPE was recorded after each round. To control for cross-contamination, a set of un-infected flasks were also prepared, and supernatant was passaged in parallel to the experimental samples. A 10-fold serial dilution of SARS-CoV-2 was also inoculated into a set of flasks starting from 1.7 × 10^7^ TCID_50_/ml and diluted to 1.1 TCID_50_/ml to show the limit of detection (LOD) of the flask passage assay and demonstrate a suitable environment for the passage and propagation of the virus.

### Statistical Analysis

All data were graphically represented and statistically analysed using GraphPad Prism 8. The Kruskal–Wallis analysis of variance (ANOVA) was performed on data sets with Dunn’s multiple comparison *post hoc*.

## Results

The inactivation of SARS-CoV-2 was assessed using three different RNA lysis buffers with and without the inclusion of a heat step. The viability of virus was determined quantitatively using the TCID_50_ assay and qualitatively by serially passaging samples in flask.

### Determination of Starting Concentration of Severe Acute Respiratory Syndrome Coronavirus-2

**Figure 1:**
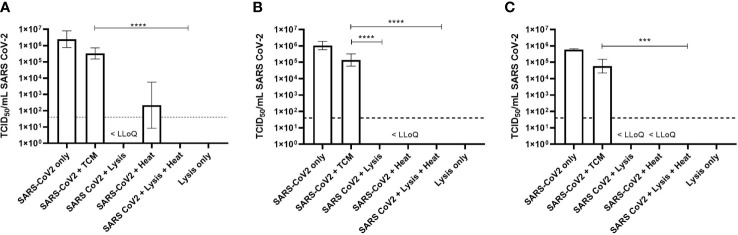
Titre of SARS-CoV-2 by TCID_50_ assay following inactivation protocols. **(A)** Qiagen protocol (Virus QIAcube HT Kit). **(B)** MagMAX Protocol 1 (Pathogen DNA/RNA). **(C)** MagMAX Protocol 2 (Viral Pathogen Kit). Geometric Mean + Geometric Standard Deviation collated from triplicate results from three separate occasions (n = 9). Dashed line = lower limit of quantification (LLoQ < 32 TCID_50_/ml); tissue culture medium (TCM); <LLoQ on graph indicates viable virus was recovered in some replicates but was below limit of quantification. Kruskal–Wallis ANOVA with Dunn’s multiple comparison *post hoc*, where ***p < 0.001, ****p < 0.0001; statistical analysis excludes virus stock and lysis only data.

These studies used the highest working concentration of SARS-CoV-2 that was available, and this ranged from 5.9 × 10^5^ to 3.5 × 10^6^ TCID_50_/ml (**Figure 1**). Following the inactivation procedure, residual toxic lysis buffer components were removed by way of multiple wash steps. Residual chemical components would otherwise be toxic to the cell-based assays. To determine if the multiple wash steps by centrifugation resulted in a loss of virus, virus was inoculated into TCM without the addition of lysis reagents (as described in the *Methods*) and assayed as described. This highlighted that there was approximately a 1-Log_10_ drop in titre in each experiment.

### Chemical Inactivation of Severe Acute Respiratory Syndrome Coronavirus-2

When virus was added to the Qiagen lysis buffer, there was a statistically significant 5-Log_10_ drop (p = 0.002) in virus titre from 3.3 × 10^5^ TCID_50_/ml to below the lower limit of quantification (LLoQ) of 32 TCID_50_/ml. Complete inactivation was not achieved however, as virus was detected below the LLoQ, but this was not quantifiable. However, by extrapolation, it was estimated that the titre was 6.2 TCID_50_/ml (**Figure 1A**).

Similar results were observed when virus was inactivated using the MagMAX Protocol 2; complete inactivation was not achieved as virus was detected below the LLoQ and was not quantifiable. The starting titre of virus for these experiments, following washing steps, was 5.8 × 10^4^ TCID_50_/ml, demonstrating a 4-Log_10_ drop in viral titre following inactivation (p < 0.001) (**Figure 1C**).

Virus inactivation following the MagMAX Protocol 1 resulted in no detectable virus by TCID_50_ assay. The starting concentration of virus, following washing steps, was calculated to be 1.4 × 10^5^ TCID_50_/ml, thus demonstrating a 5-Log_10_ drop in viral titre with this particular protocol (p < 0.0001) (**Figure 1B**).

### Heat Inactivation of Severe Acute Respiratory Syndrome Coronavirus-2

Heat alone or in combination with lysis buffer was also investigated as a means to inactivate SARS-CoV-2. For each experiment, virus in TCM was heated at 68°C for 15 min and centrifuged to maintain consistency with samples in lysis buffer. Although not statistically significant, at least a 3-Log_10_ drop in viral titre was observed following heat treatment alone, though viable virus was observed in replicates across all three heat alone experiments, even when below LLoQ (**Figure 1**).

When the virus was added to one of the three lysis buffers and subsequently heated, no viable virus was detected following TCID_50_ assay and an average drop in viral titre of 5-Log_10_ across all experiments (p < 0.0001) (**Figures 1A–C**).

### Confirmation of Inactivation by Viral Propagation

To confirm findings by TCID_50_ assay, viral samples were propagated in cell culture flasks over a total of three passages to identify potential viral breakthrough. **Table 2** shows the results of the presence of CPE after the first passage. The LOD for viral propagation was determined following propagation of serially diluted virus stocks (**Table 2** row 1 to 5), and on average, the LOD was 1.3 TCID_50_/ml.

**Table 2:** Summary of results following cell culture passage and TCID_50_ assay.

Inactivation protocol	Qiagen protocol		MagMAX Protocol 1		MagMAX Protocol 2	
	(96 Virus QIAcube HT Kit)		(Pathogen DNA/RNA Kit)		(Viral Pathogen Kit)	
Sample description	Flasks infected/total flasks	TCID_50_/ml (SD)	Flasks infected/total flasks	TCID_50_/ml (SD)	Flasks infected/total flasks	TCID_50_/ml (SD)
1. SARS-CoV-2 starting titre	3/3	1.7 × 10^7^	3/3	5.9 × 10^6^	3/3	3.0 × 10^6^
2. SARS-CoV-2 10^−4^ dilution	3/3	1.7 × 10^3^*	3/3	5.9 × 10^2^*	3/3	3.0 × 10^2^*
3. SARS-CoV-2 10^−5^ dilution	3/3	1.7 × 10^2^*	3/3	59.4*	2/3	20.0*
4. SARS-CoV-2 10^−6^ dilution	3/3	17*	1/3	2.0*	1/3	0.7*
5. SARS-CoV-2 10^−7^ dilution	2/3	1.1*	0/3	ND	0/3	ND
6. SARS-CoV-2 + TCM	9/9	3.3 × 10^5^	9/9	1.4 × 10^5^	9/9	5.8 × 10^4^
7. SARS-CoV-2 + lysis buffer	3/9	<LLoQ	0/9	ND	0/9	<LLoQ
8. SARS-CoV-2 + heat	9/9	2.2 × 10^2^	9/9	29.6	8/9	20.4
9. SARS-CoV-2 + lysis buffer + heat	0/9	ND	0/9	ND	0/9	ND
10. TCM + lysis buffer	0/9	ND	0/0	ND	0/9	ND

Passage results shown are after the third serial. TCID_50_ titres are geometric mean titre/ml. <LLoQ indicates viable virus was recovered in some replicates but was below limit of quantification.

SARS-2, SARS-CoV-2; TCM, tissue culture medium; LLoQ, lower limit of quantification (10 TCID_50_/ml); SD, standard deviation; ND, not detected.

*Indicates the TCID_50_/ml is extrapolated from performing 10-fold dilutions from a known starting concentration and calculated based on number of flasks infected.

When virus was added to TCM, CPE was present in all flasks as expected (**Table 2** row 6, positive control). No cell toxicity was observed from negative control samples where TCM only was added to lysis buffer and washed as described previously (**Table 2** row 10, negative control).

When SARS-CoV-2 was inactivated following the Qiagen protocol, three out of the nine flasks were scored as positive for CPE. Of the flasks where no CPE was observed, no breakthrough of virus was seen as a result of serial passage (**Table 2** row 7). These data align with the TCID_50_ assays, where Qiagen lysis buffer alone did not completely inactivate the virus. Following both MagMAX protocols, zero out of the nine flasks were scored positively for CPE (**Table 2** row 7). For the MagMAX Protocol 1, this confirms the TCID_50_ results, where no viable virus was also observed. For the MagMAX Protocol 2, virus was detected but not quantifiable in the TCID_50_ assay (below the LLoQ); however, subsequent serial passage did not provide evidence of viability, as all flasks were negative for CPE.

When SARS-CoV-2 was added to TCM and heated for 15 min at 68°C, CPE was observed in all but one flask (**Table 2** row 8), confirming the TCID_50_ results that the heating protocol described here does not completely inactivate the virus.

For all inactivation protocols, when SARS-CoV-2 samples were treated in a two-step manner (lysis buffer and heat), no viable virus was detected in either the quantitative or qualitative assays (**Figure 1** and **Table 2** row 9). These data provide strong evidence that the lysis buffers described here in combination with the heat protocol can completely inactivate up to 5-Log_10_ TCID_50_/ml SARS-CoV-2.

## Discussion

Real-time PCR is the gold standard clinical diagnostic method for the detection of SARS-CoV-2 in patients displaying symptoms of COVID-19. There has been a rapid development in RNA extraction and RT-PCR diagnostic methods in order to help prevent further spread of infection through communities. It is crucial that testing is accurate and efficient, both of which must not compromise safety of those processing the samples (Dhamad and Abdal Rhida, 2020). Laboratory-acquired infections due to incomplete inactivation or incorrect handling of samples have been reported for SARS-CoV (Lim et al., 2004; Taylor et al., 2005) as well as many other infectious agents (Singh, 2009).

To date, there are only a handful of publications reporting the use of nucleic acid isolation reagents, detergents, and heat to inactivate SARS-CoV-2 (Kim et al., 2020; Pastorino et al., 2020; Welch et al., 2020; Burton et al., 2021); and due to commercial sensitivity, manufacturers of extraction kits are not required to publish the full ingredient list of proprietary buffers [with potential viral inactivating components only inferred if they are listed on associated Material Safety Data Sheets (MSDSs)], and post-treatment viability test methods vary in stringency across studies.

In our study, we investigated the SARS-CoV-2 inactivation efficacy of viral lysis buffers from three commercially available kits developed to allow RNA extraction on high-throughput (96-well) automated platforms. Stringent post-treatment assessments of viral viability were then conducted. For each kit, the initial lysis buffer mix, developed from manufacturer’s instructions, included a guanidine-based lysis buffer with additional viral inactivating components such as a solvent and/or a detergent. Each mix was added to 200 µl of a representative clinical sample (SARS-CoV-2 in VTM). Furthermore, we tested all three protocols with and without the addition of a thermal inactivation step at 68°C for 15 min.

We started with the highest possible titre of SARS-CoV-2 that we had available and first determined the titre of virus following wash steps, which were required to remove any chemical compounds that would be cytotoxic to the cell-based assays. We chose to remove the reagents from the samples by centrifugation and, in doing so, demonstrated a loss of approximately 1-Log_10_ of virus. Other researchers have used centrifugation columns or filters but again report a similar loss in viral titre (Patterson et al., 2020) or residual toxicity leading to reduced sensitivity of the read-out of the assays (Welch et al., 2020). The wash steps employed here eliminated all residual toxicity, allowing the sensitivity of our assay read-outs to be unaffected.

In our study, the chemicals used to assess the inactivation of SARS-CoV-2 were combinations of GITC, detergent, and solvent. The Qiagen protocol (using reagents from the QIAamp 96 Virus QIAcube HT Kit) and the MagMAX Protocol 2 (using reagents from the MagMAX viral/pathogen nucleic acid isolation kit) both included GITC and a detergent (SDS or Zwittergent, respectively) (**Table 1**). Both of these inactivation buffers significantly reduced viral titres of SARS-CoV-2 by 4-Log_10_; however, complete inactivation of viable virus was not achieved, as detectable, but not quantifiable, virus was detected in the TCID_50_ assay (below LLoQ). Subsequent serial passage of viral samples following inactivation using the Qiagen protocol demonstrated virus breakthrough, confirming the results observed in the TCID_50_ assay. It was also anticipated that serial passage of virus inactivated following MagMAX Protocol 2 would have amplified and enabled virus breakthrough too, but this was not observed. The stated GITC composition of Qiagen Buffer ACL (30%–50%) is lower than that of the MagMAX Lysis buffer (55%–80%), and thus, the higher GITC composition in the MagMAX buffer may have exerted a greater efficacy of viral inactivation, although we could not demonstrate complete inactivation. As described previously, GITC-based chemicals alone have been reported to inactivate some viruses (Blow et al., 2004; Ngo et al., 2017); but as observed here and by others, this is not always the case (Kumar et al., 2015; Smither et al., 2015; Burton et al., 2017). Studies by Pastorino et al. (2020) have assessed the inactivation of SARS-CoV-2 using the detergent containing Buffer ATL and, in contrast to our findings, reported greater than a 6-Log_10_ drop in virus titre. The SDS composition of Buffer ATL used by Pastorino et al. (2020) was 1%–10%; however, the SDS composition of ATL buffer in our study was 1% to <3% SDS (**Table 1**). Pastorino et al. (2020) also used a 1:1 ratio of ATL buffer to sample, where as in our protocol we used a reagent-to-sample ratio of 0.5:1. Thus, the work of Pastorino et al. (2020) infers a higher concentration of this detergent, and larger reagent-to-sample ratio would be critical for the inactivation process. This also underlines the potential for different concentrations of components in products that are ostensibly the same. Patterson et al. (2020) and Welch et al. (2020) screened a number of detergents for their inactivation efficacy against SARS-CoV-2. Patterson et al. (2020) reported that 0.5% SDS inactivated SARS-CoV-2 but used a low starting titre of 10^2^ PFU (Patterson et al., 2020), whereas Welch et al. (2020) also reported a drop in virus titre of 6.5-Log_10_ TCID_50_/ml, but viable virus was still observed (Welch et al., 2020).

In our study, the only protocol that inactivated virus without an additional heat step was MagMAX Protocol 1 (using reagents from the MagMAX Pathogen RNA/DNA kit), where no CPE was observed from either TCID_50_ assay or following three rounds of serial passage in tissue culture flasks. The MagMAX Protocol 1 included the MagMAX lysis binding buffer that contained GITC and the detergent Zwittergent. With the addition of 2-propanol within the lysis buffer mix, there were, therefore, three components likely to exert a disruptive effect on the SARS-CoV-2 viral envelope. The reagent-to-sample ratio of 3.8:1 was also higher, with more than double the volume of lysis buffer mix added to each sample, compared with the other two methods assessed (**Table 1**).

Our results suggest that both a high reagent-to-sample ratio and the incorporation of a solvent improved the inactivation efficacy of a chemical only method. The SARS-CoV-2 inactivation efficacy of the GITC-based Buffer AVL (Qiagen) in combination with ethanol has been assessed in two studies. Complete SARS-CoV-2 inactivation was reported by Welch et al. (2020) in contrast to incomplete inactivation by Pastorino et al. (2020). This contradiction in findings could be due to the ratios of reagent, solvent, and sample used. Both studies used 4 volumes of AVL to 1 volume of sample; however, volumes of ethanol used in combination with Buffer AVL may explain the varying results. Welch et al. (2020) used 4 volumes of ethanol in combination with AVL and sample, whereas Pastorino et al. (2020) only added 1 volume of ethanol to the AVL–sample combination. In our studies using the MagMAX Protocol 1, the ratio of lysis buffer and isopropanol was considerably less with 1.8 volumes of lysis buffer and 1.5 volumes of solvent, but the addition of the detergent Zwittergent (within the MagMAX Lysis Buffer) may have enhanced the inactivation. The addition of the enzyme Proteinase K in both the Qiagen method and MagMAX Protocol 2 (which was absent in MagMAX Protocol 1) did not appear to have enhanced inactivation efficacy.

We also investigated the efficacy of thermal inactivation, by heating the sample to, and then maintaining at, 68°C for 15 min. Heat inactivation alone reduced the viral titre by 3-Log_10_, although this was not statistically significant compared with the controls and was not as effective as the use of lysis buffers alone. Burton et al. (2021) reported similar findings with incomplete inactivation of SARS-CoV-2 at 56°C and 60°C for up to 60 min. In contrast, some studies have reported the successful use of heat for complete inactivation of SARS-CoV and SARS-CoV-2 (Darnell et al., 2004; Kim et al., 2020). Kim et al. (2020) demonstrated the complete inactivation of SARS-CoV-2 in clinical samples following incubation at 65°C for 30 min, although this work was based on quantitative TCID_50_ assays alone. Furthermore, Darnell et al. (2004) reported complete inactivation of SARS-CoV after heating at 65°C for 60 min; longer time was required to ensure any viral aggregates were fully exposed and inactivated by the heat treatment.

The use of heat to inactivate virus has been reported to reduce viral RNA stability (Pan et al., 2020; Zou et al., 2020); and depending on the target gene used for RT-PCR, incubation at 65°C for 30 min can significantly reduce the target copy numbers, leading to false-negative results of clinical samples (Kim et al., 2020; Zou et al., 2020). The DCL has an accredited SARS-CoV-2 diagnostic workflow (UKAS, 2020) using the Qiagen and KingFisher (using MagMAX Protocol 1) extraction platforms each with an additional heat inactivation step. Multiple External Quality Assessment panels and reference standards have been tested during DCL set-up and operation. The E-Gene PCR assay (Corman et al., 2020) is used in this laboratory, and in our hands, the heat inactivation regime we employ does not appear to adversely affect PCR results.

In determining the practical relevance of our work, the viral loads in COVID-19 samples likely to be encountered in a high-throughput diagnostic laboratory should be considered. Currently, there is little information on the infectious viral load present on a clinical nasal/throat swab. Most studies only report quantification cycle (C_q_) values following RT-PCR (Pan et al., 2020), but one study has estimated that there is a median titre of 10^3^ TCID_50_/ml collected from 90 nasopharyngeal or endotracheal clinical samples (Bullard et al., 2020). During DCL validation studies, a precisely defined reference standard dilution series of entire SARS-CoV-2 virions (SARS-CoV-2 Analytical Q Panel; Qnostics Ltd, United Kingdom) was tested (data not shown). Within this series, the highest concentration of material was 6-Log_10_ digital copies (dC)/ml; and following RNA extraction using the Qiagen method described in this paper, mean E-gene (Corman et al., 2020) quantification cycle (C_q_) values of 22.65 were returned from this concentration. During DCL operations, we have commonly tested positive samples with E-gene PCR C_q_ values in teens, with occasional samples returning C_q_ values <13. Although care must be taken in comparing and extrapolating PCR (C_q_), TCID_50_/ml, and dC/ml values, this is consistent with a study reporting similarly low C_q_ values from COVID patients early in the infection cycle (Jang et al., 2021) and indicates that some swab samples can contain very high viral loads.

We have demonstrated the SARS-CoV-2 inactivation efficacy of the reagents found in lysis buffers of three commercially available kits used on high-throughput extraction platforms. Only when combined with a heat step did all methods show a complete inactivation of SARS-CoV-2 by both TCID_50_ assay and by sequential passage in tissue culture. Therefore, in the DCL, samples are sequentially mixed with lysis buffer and then followed with heat treatment. This approach also extends the contact time of lysis buffer to sample, which should further enhance the inactivation efficacy of the buffers and mitigates the fact that in this inactivation study we were unable to test samples with a starting concentration greater than 5.8 × 10^5^ TCID_50_/ml (in view of the likely higher concentrations seen in samples received). In our studies, we also did not include samples that contain potential interfering substances or true samples; however, Pastorino et al. (2020) did include interfering substances and a range of clinical samples, and no obvious impact of these sample types was reported on the efficacy of the viral inactivation process.

Due to the contrasting literature for inactivation of SARS-CoV-2 (and that of viruses generally), a case-by-case assessment of different inactivation protocols is essential to prevent laboratory-acquired infections. To ensure the highest safety standards (and also taking into account the high viral loads of samples tested), in the operational DCL, we employ methods that utilise the inactivation efficacies of the chemical components of lysis buffers found in commercial kits with that of the heat. As a result, the high-throughput RNA extraction platforms are performed on the open bench rather than within a Class 1 microbiological safety cabinet. All laboratories must make the appropriate assessments regarding methods applicable to their unique set of circumstances. The results presented in this study may help laboratories undertake such assessments, especially if they do not have access to high containment facilities to complete in-house inactivation studies.

## Data Availability Statement

The original contributions presented in the study are included in the article/supplementary material. Further inquiries can be directed to the corresponding author.

## Author Contributions

RT: Conceptualisation, data curation, formal analysis, investigation, methodology, validation, writing—original draft, and writing—review and editing. LE: Data curation, investigation, methodology, and writing—review and editing. LO’B: Data curation, investigation, methodology, and writing—review and editing. DU: Data curation, investigation, methodology, and writing—review and editing. JF: Investigation, methodology, and writing—review and editing. SS: Data curation, formal analysis, investigation, methodology, and writing—review and editing. AP: Data curation, investigation, methodology, and writing—review and editing. HS: Methodology, resources, and writing—review and editing. KH: Conceptualisation, writing—original draft, and writing—review and editing. SW: Conceptualisation, funding acquisition, methodology, project administration, validation, writing—original draft, and writing—review and editing. All authors contributed to the article and approved the submitted version.

## Funding

The set-up and validation of the Defence COVID Laboratory (of which this study was a part) was funded by the UK Department of Health and Social Care (DHSC).

## Conflict of Interest

The authors declare that the research was conducted in the absence of any commercial or financial relationships that could be construed as a potential conflict of interest.

## Publisher’s Note

All claims expressed in this article are solely those of the authors and do not necessarily represent those of their affiliated organizations, or those of the publisher, the editors and the reviewers. Any product that may be evaluated in this article, or claim that may be made by its manufacturer, is not guaranteed or endorsed by the publisher.

## References

[B1] Blow J. A. Dohm D. J. Negley D. L. Mores C. N. (2004). Virus Inactivation by Nucleic Acid Extraction Reagents. J. Virol. Methods 119, 195–198. doi: 10.1016/j.jviromet.2004.03.015 15158603

[B2] Bullard J. Dust K. Funk D. Strong J. E. Alexander D. Garnett L.. (2020). Predicting Infectious SARS-CoV-2 From Diagnostic Samples. Clin. Infect. Dis. 71, 2663–2666. doi: 10.1093/cid/ciaa638 32442256 PMC7314198

[B3] Burton J. E. Easterbrook L. Pitman J. Anderson D. Roddy S. Bailey D.. (2017). The Effect of a non-Denaturing Detergent and a Guanidinium-Based Inactivation Agent on the Viability of Ebola Virus in Mock Clinical Serum Samples. J. Virol. Methods 250, 34–40. doi: 10.1016/j.jviromet.2017.09.020 28941617

[B4] Burton J. Love H. Richards K. Burton C. Summers S. Pitman J.. (2021). The Effect of Heat-Treatment on SARS-CoV-2 Viability and Detection. J. Virol. Methods 290, 114087. doi: 10.1016/j.jviromet.2021.114087 33515663 PMC7840429

[B5] Chen J. (2020). Pathogenicity and Transmissibility of 2019-Ncov-A Quick Overview and Comparison With Other Emerging Viruses. Microbes Infect. 22, 69–71. doi: 10.1016/j.micinf.2020.01.004 32032682 PMC7102641

[B6] Corman V. M. Landt O. Kaiser M. Molenkamp R. Meijer A. Chu D. K. W.. (2020). Detection of 2019 Novel Coronavirus (2019-Ncov) by Real-Time RT-PCR. Eurosurveillance 25, 23–30. doi: 10.2807/1560-7917.ES.2020.25.3.2000045 PMC6988269 31992387

[B7] Darnell M. E. Subbarao K. Feinstone S. M. Taylor D. R. (2004). Inactivation of the Coronavirus That Induces Severe Acute Respiratory Syndrome, SARS-CoV. J. Virol. Methods 121, 85–91. doi: 10.1016/j.jviromet.2004.06.006 15350737 PMC7112912

[B8] Dhamad A. E. Abdal Rhida M. A. (2020). COVID-19: Molecular and Serological Detection Methods. PeerJ 8, e10180. doi: 10.7717/peerj.10180 33083156 PMC7547594

[B9] Gorbalenya A. E. Baker S. C. Baric R. S. de Groot R. J. Drosten C. Gulyaeva A. A.. (2020). The Species Severe Acute Respiratory Syndrome-Related Coronavirus: Classifying 2019-Ncov and Naming it SARS-CoV-2. Nat. Microbiol. 5, 536–544. doi: 10.1038/s41564-020-0695-z 32123347 PMC7095448

[B10] Jang S. Rhee J. Y. Wi Y. M. Jung B. K. (2021). Viral Kinetics of SARS-CoV-2 Over the Preclinical, Clinical, and Postclinical Period. Int. J. Infect. Dis. 102, 561–565. doi: 10.1016/j.ijid.2020.10.099 33160066 PMC7642732

[B11] Kim Y. I. Casel M. A. B. Kim S. M. Kim S. G. Park S. J. Kim E. H.. (2020). Development of Severe Acute Respiratory Syndrome Coronavirus 2 (SARS-CoV-2) Thermal Inactivation Method With Preservation of Diagnostic Sensitivity. J. Microbiol. 58, 886–891. doi: 10.1007/s12275-020-0335-6 32989642 PMC7522010

[B12] Kumar M. Mazur S. Ork B. L. Postnikova E. Hensley L. E. Jahrling P. B.. (2015). Inactivation and Safety Testing of Middle East Respiratory Syndrome Coronavirus. J. Virol. Methods 223, 13–18. doi: 10.1016/j.jviromet.2015.07.002 26190637 PMC4555185

[B13] Leclercq I. Batejat C. Burguiere A. M. Manuguerra J. C. (2014). Heat Inactivation of the Middle East Respiratory Syndrome Coronavirus. Influenza Other Respir. Viruses 8, 585–586. doi: 10.1111/irv.12261 25074677 PMC4181824

[B14] Lim P. L. Kurup A. Gopalakrishna G. Chan K. P. Wong C. W. Ng L. C.. (2004). Laboratory-Acquired Severe Acute Respiratory Syndrome. N Engl. J. Med. 350, 1740–1745. doi: 10.1056/NEJMoa032565 15103000

[B15] Mayo D. R. Beckwith W. H. (2002). Inactivation of West Nile Virus During Serologic Testing and Transport. J. Clin. Microbiol. 40, 3044–3046. doi: 10.1128/JCM.40.8.3044-3046.2002 12149375 PMC120620

[B16] Ngo K. A. Jones S. A. Church T. M. Fuschino M. E. George K. S. Lamson D. M.. (2017). Unreliable Inactivation of Viruses by Commonly Used Lysis Buffers. Appl. Biosafety 22, 56–59. doi: 10.1177/1535676017703383

[B17] Pan Y. Long L. Zhang D. Yuan T. Cui S. Yang P.. (2020). Potential False-Negative Nucleic Acid Testing Results for Severe Acute Respiratory Syndrome Coronavirus 2 From Thermal Inactivation of Samples With Low Viral Loads. Clin. Chem. 66, 794–801. doi: 10.1093/clinchem/hvaa091 32246822 PMC7184485

[B18] Pan Y. Zhang D. Yang P. Poon L. L. M. Wang Q. (2020). Viral Load of SARS-CoV-2 in Clinical Samples. Lancet Infect. Dis. 20, 411–412. doi: 10.1016/S1473-3099(20)30113-4 32105638 PMC7128099

[B19] Pastorino B. Touret F. Gilles M. Luciani L. de Lamballerie X. Charrel R. N. (2020). Evaluation of Chemical Protocols for Inactivating SARS-CoV-2 Infectious Samples. Viruses-Basel 12, 624. doi: 10.3390/v12060624 PMC7354533 32521706

[B20] Patel R. Babady E. Theel E. S. Storch G. A. Pinsky B. A. St George K.. (2020). Report From the American Society for Microbiology COVID-19 International Summit, 23 March 2020: Value of Diagnostic Testing for SARS-CoV-2/COVID-19. mBio 11, e00722–20. doi: 10.1128/mBio.00722-20 32217609 PMC7157705

[B21] Patterson E. I. Prince T. Anderson E. R. Casas-Sanchez A. Smith S. L. Cansado-Utrilla C.. (2020). Methods of Inactivation of SARS-CoV-2 for Downstream Biological Assays. J. Infect. Dis. 222, 1462–1467. doi: 10.1093/infdis/jiaa507 32798217 PMC7529010

[B22] Piercy T. J. Smither S. J. Steward J. A. Eastaugh L. Lever M. S. (2010). The Survival of Filoviruses in Liquids, on Solid Substrates and in a Dynamic Aerosol. J. Appl. Microbiol. 109, 1531–1539. doi: 10.1111/j.1365-2672.2010.04778.x 20553340

[B23] Public Health England (2020) COVID-19: Guidance for Sampling and for Diagnostic Laboratories. Available at: https://wwwgovuk/government/publications/wuhan-novel-coronavirus-guidance-for-clinical-diagnostic-laboratories.

[B24] Reed L. J. Muench H. (1938). A Simple Method of Estimating Fifty Per Cent Endpoints. Am. J. Epidemiol. 27, 493–497. doi: 10.1093/oxfordjournals.aje.a118408

[B25] Shental N. Levy S. Wuvshet V. Skorniakov S. Shalem B. Ottolenghi A.. (2020). Efficient High-Throughput SARS-CoV-2 Testing to Detect Asymptomatic Carriers. Sci. Adv. 6, eabc5961. doi: 10.1126/sciadv.abc5961 32917716 PMC7485993

[B26] Singh K. (2009). Laboratory-Acquired Infections. Clin. Infect. Dis. 49, 142–147. doi: 10.1086/599104 19480580 PMC7107998

[B27] Smither S. Phelps A. Eastaugh L. Ngugi S. O'Brien L. Dutch A.. (2016). Effectiveness of Four Disinfectants Against Ebola Virus on Different Materials. Viruses 8, 185. doi: 10.3390/v8070185 PMC4974520 27399759

[B28] Smither S. J. Weller S. A. Phelps A. Eastaugh L. Ngugi S. O'Brien L. M.. (2015). Buffer AVL Alone Does Not Inactivate Ebola Virus in a Representative Clinical Sample Type. J. Clin. Microbiol. 53, 3148–3154. doi: 10.1128/JCM.01449-15 26179307 PMC4572529

[B29] Tahamtan A. Ardebili A. (2020). Real-Time RT-PCR in COVID-19 Detection: Issues Affecting the Results. Expert Rev. Mol. Diagn. 20, 453–454. doi: 10.1080/14737159.2020.1757437 32297805 PMC7189409

[B30] Taylor J. W. D. Bernard K. A. Masters P. S. Trimarchi C. V. (2005). SARS Coronaviruses and Highly Pathogenic Influenza Viruses: Safety and Occupational Health for Laboratory Workers. Emerg. Infect. Dis. 11, e3. doi: 10.3201/eid1104.041304

[B31] UKAS (2020) Schedule of Accreditation Issued by United Kingdom Accreditation Service. Available at: https://wwwukascom/wp-content/uploads/schedule_uploads/00002/1886Testing-Singlepdf.

[B32] van Kampen J. J. A. Tintu A. Russcher H. Fraaij P. L. A. Reusken C. B. E. M. Rijken M.. (2017). Ebola Virus Inactivation by Detergents Is Annulled in Serum. J. Infect. Dis. 216, 859–866. doi: 10.1093/infdis/jix401 28961947 PMC5853228

[B33] Welch S. R. Davies K. A. Buczkowski H. Hettiarachchi N. Green N. Arnold U.. (2020). Analysis of Inactivation of SARS-CoV-2 by Specimen Transport Media, Nucleic Acid Extraction Reagents, Detergents, and Fixatives. J. Clin. Microbiol. 58, e01713–20. doi: 10.1128/JCM.01713-20 32839250 PMC7587104

[B34] World Health Organization (2020) Laboratory Biosafety Guidance Related to Coronavirus Disease (COVID-19). WHO/WPE/GIH/20203. Available at: https://www.who.int/publications/i/item/laboratory-biosafety-guidance-related-to-coronavirus-disease-(covid-19).

[B35] Wu F. Zhao S. Yu B. Chen Y. M. Wang W. Song Z. G.. (2020). A New Coronavirus Associated With Human Respiratory Disease in China. Nature 579, 265–269. doi: 10.1038/s41586-020-2008-3 32015508 PMC7094943

[B36] Zou J. Zhi S. Chen M. Su X. Kang L. Li C.. (2020). Heat Inactivation Decreases the Qualitative Real-Time RT-PCR Detection Rates of Clinical Samples With High Cycle Threshold Values in COVID-19. Diagn. Microbiol. Infect. Dis. 98, 115109. doi: 10.1016/j.diagmicrobio.2020.115109 32593875 PMC7289114

