# Corrigendum: Evaluation of the SARS-CoV-2 Inactivation Efficacy Associated With Buffers From Three Kits Used on High-Throughput RNA Extraction Platforms

**DOI:** 10.3389/fcimb.2021.813442

**Published:** 2021-12-08

**Authors:** Ruth E. Thom, Lin S. Eastaugh, Lyn M. O’Brien, David O. Ulaeto, James S. Findlay, Sophie J. Smither, Amanda L. Phelps, Helen L. Stapleton, Karleigh A. Hamblin, Simon A. Weller

**Affiliations:** CBR Division, Dstl Porton Down, Salisbury, United Kingdom

**Keywords:** SARS-CoV-2, high throughput, PCR, biosafety, laboratory-acquired infection, clinical diagnosis

In the original article, there was a mistake in **Table 1** *: Protocols tested for assessing inactivation using lysis buffers* as published. During the publication process the components for each of the three kits tested in this study (as stated in the ‘Reagents’ and ‘Active virucidal components’ columns), were unclearly formatted. The corrected **Table 1** *: Protocols tested for assessing inactivation using lysis buffers* appears below.

**Table 1:** Protocols tested for assessing inactivation using lysis buffers.

Manufacturer, RNA extraction kit, Platform	Reagents (volume/sample)	Active virucidal components*	Reagent: Sample ratio
Qiagen, QIAamp 96 Virus QIAcube HT Kit (Cat #: 57731), Qiagen Qiacube HT. *(Referred to here as Qiagen protocol)*	ACL buffer (190 µl)	GITC 30 - <50%	1.6: 1
	ATL buffer (100 µl)	1 - <3% SDS	
	Proteinase K (20 µl)		
	Carrier RNA (5 µl)		
	MS2 (10 µl)		
ThermoFisher, MagMax Pathogen RNA/DNA kit (Cat #: 4462359), Kingfisher Flex. *(Referred to here as MagMax Protocol 1)*	Lysis binding buffer (350 µl)	GITC 55-80% <0.001% Acrylamide Zwittergent	3.8: 1
	Isopropanol (300 µl)	100% 2-propanol	
	Carrier RNA (2 µl)		
	Water (100 µl)		
	MS2 (10 µl)		
ThermoFisher, MagMax viral/pathogen nucleic acid isolation kit (Cat #: A48310), Kingfisher Flex. *(Referred to here as MagMax Protocol 2)*	Lysis binding buffer (265 µl)	GITC 55-80% <0.001% Acrylamide Zwittergent	1.4: 1
	Proteinase K (5 µl)		
	^†^Water (Magnetic beads) (10 µl)		
	MS2 (10 µl)		

*As identified directly from components, manufacturer information, or inferred from the associated MSDS.

^†^Water was used to replace the magnetic beads as the washing steps described below would not remove the beads and the beads interfered the read-out of the TCID-_50_ assay.

GITC, Guanidinium thiocyanate; SDS, Sodium dodecyl sulphate.

The authors apologize for this error and state that this does not change the scientific conclusions of the article in any way. The original article has been updated.

## Publisher’s Note

All claims expressed in this article are solely those of the authors and do not necessarily represent those of their affiliated organizations, or those of the publisher, the editors and the reviewers. Any product that may be evaluated in this article, or claim that may be made by its manufacturer, is not guaranteed or endorsed by the publisher.

